# Maturation of Intestinal Oxygenation: A Review of Mechanisms and Clinical Implications for Preterm Neonates

**DOI:** 10.3389/fped.2020.00354

**Published:** 2020-07-03

**Authors:** Baukje M. Dotinga, Jonathan P. Mintzer, James E. Moore, Jan B. F. Hulscher, Arend F. Bos, Elisabeth M. W. Kooi

**Affiliations:** ^1^Division of Neonatology, Department of Pediatrics, Beatrix Children's Hospital, University Medical Center Groningen, University of Groningen, Groningen, Netherlands; ^2^Division of Neonatal-Perinatal Medicine, Department of Pediatrics, Mountainside Medical Center, Montclair, NJ, United States; ^3^Division of Neonatal-Perinatal Medicine, Department of Pediatrics, Connecticut Children's Medical Center, University of Connecticut School of Medicine, Hartford, CT, United States; ^4^Division of Pediatric Surgery, Department of Surgery, University Medical Center Groningen, Groningen, Netherlands

**Keywords:** newborn, preterm neonates, intestinal circulation, intestinal oxygenation, near-infrared spectroscopy, growth and development

## Abstract

Nutrient requirements of preterm neonates may be substantial, to support growth and maturation processes in the presence of challenging post-natal circumstances. This may be accompanied by substantial intestinal oxygen requirements. Preterm neonates may not be able to meet these oxygen requirements, due to a developmental delay in intestinal oxygenation regulation mechanisms. This review summarizes the available literature on post-natal maturation of intestinal oxygenation mechanisms and translates these changes into clinical observations and potential implications for preterm neonates. The different mechanisms that may be involved in regulation of intestinal oxygenation, regardless of post-natal age, are first discussed. The contribution of these mechanisms to intestinal oxygenation regulation is then evaluated in newborn and mature intestine. Finally, the course of clinical observations is used to translate these findings to potential implications for preterm neonates.

## Introduction

The major functions of the intestine are immunological and digestion-absorption (1, 2). The immunological function includes a wide variety of cells and strategies, beyond the scope of this review, and among other things prevents bacterial translocation across the epithelium in the presence of microbial colonization of the gut (1, 3, 4). The digestive-absorptive function includes production of digestive enzymes and absorption of carbohydrates, lipids, proteins, and vitamins (2). Ultimately, the intestine provides required nutrients to support body growth and function. The nutrient requirements of neonates may be substantial, consequent to maturation processes and tissue growth (5, 6). In preterm neonates, these nutritional requirements may be even greater, due to challenging post-natal circumstances, e.g., infection and respiratory distress (7). To meet ongoing nutritional demands, sufficient intestinal oxygenation is essential for intestinal function (8).

After birth, the intestine transitions from a relatively dormant organ to the sole site for nutrient absorption, thus requiring a concomitant increase in oxygen supply (9). This transition is accompanied by rapid tissue growth relative to the whole body, as the intestine increases its weight by 40–70% within 24 h and 4-fold within 10 days (10). Additionally, the change from continuously swallowing amniotic fluid to tolerating intermittent enteral feeds may alter intestinal physiology (11). Therefore, the intestine and its associated circulation are subject to a considerable fetal-to-neonatal adaptation which continues to mature during early life (9, 11). Although little is known about post-natal changes in the intestinal circulation of preterm neonates, it can be speculated that these occur with increasing gestational age and post-natal age, as was recently described for the cerebral circulation (12). Among other factors, this may predispose preterm neonates to gastrointestinal complications, such as feeding issues, necrotizing enterocolitis (NEC), and poor growth (13–15).

Monitoring of intestinal oxygenation may facilitate early identification of gastrointestinal complications. Intestinal blood flow velocity can be measured using Doppler, but this provides only momentary information on oxygen delivery and not oxygen consumption (16). Near-infrared spectroscopy (NIRS) is a non-invasive, bedside technique used to continuously monitor regional oxygen saturation (rSO_2_) (17). Previous studies have reported promising results for splanchnic rSO_2_ (r_s_SO_2_)-monitoring for detecting hemodynamic changes that accompany physiologic and pathophysiologic conditions (18, 19). Currently, the use of splanchnic NIRS remains mostly limited to research settings. A better understanding of the different mechanisms regulating intestinal oxygenation may facilitate interpretation of r_s_SO_2_ and advance clinical applications of r_s_SO_2_-monitoring.

In this review, we will first discuss the presumed mechanisms regulating intestinal oxygenation regardless of post-natal age, derived primarily from animal models. Next, we will discuss maturation of these mechanisms and elaborate on potential clinical implications for preterm neonates.

## Overview of Mechanisms Regulating Intestinal Oxygenation

Intestinal oxygenation represents the balance between oxygen supply and oxygen demand. Intestinal oxygen supply can be divided into convective delivery, from mesenteric arteries to intestinal capillaries, and diffusive delivery, from intestinal capillaries to parenchymal cells (20). Convective delivery depends on intestinal blood flow and arterial oxygen content and is modulated by resistance vessels, i.e., terminal mesenteric and submucosal arterioles (20, 21). In contrast, diffusive delivery depends on functional capillary density and capillary-to-cell local PO_2_ gradients, and is modulated by precapillary sphincters (20, 21).

**Figure 1:**
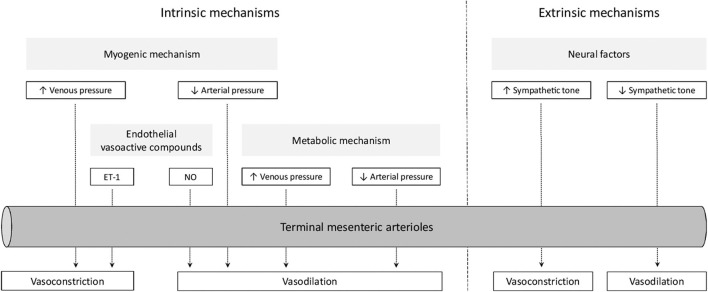
Overview of mechanisms regulating intestinal oxygenation. ET-1, endothelin-1; NO, nitric oxide.

Intestinal oxygenation is regulated at both local and systemic levels (21, 22). Local mechanisms provide real-time modulation of intestinal oxygenation and reflect an intestinal intrinsic capacity (21, 23). Systemic, or extrinsic, mechanisms integrate intestinal circulation into the systemic circulation (22). In the next sections, we will discuss these mechanisms in more detail. These mechanisms are summarized in Figure 1.

### Intrinsic Regulation of Intestinal Oxygenation

Intrinsic mechanisms for regulating intestinal oxygenation are divided into myogenic factors, metabolic factors, and endothelial vasoactive compounds (24). Both myogenic and metabolic factors participate in pressure-flow autoregulation, i.e., the ability to maintain vascular flow during changes in perfusion pressure. However, the contribution of metabolic factors to this phenomenon seem to exceed those of myogenic factors (25). Additionally, overall vascular responses may be modified by endothelial vasoactive compounds (26).

The myogenic mechanism aims to sustain capillary pressure and transcapillary fluid exchange during changes in transmural pressure (23, 27). Increases in intravascular pressure lead to vasoconstriction in resistance vessels and closure of precapillary sphincters (28). Myogenic vasoconstriction in response to circumferential stretch of vascular smooth muscle is mediated by Ca^2+^ influx, Ca^2+^ release from the sarcoplasmic reticulum, and increased Ca^2+^-sensitivity of contractile myofilaments (29). Intracellular signaling pathways leading to these events may involve protein kinase C (30).

The metabolic mechanism aims to sustain blood flow and oxygen delivery during changes in tissue metabolism (23). Increases in tissue metabolism leads to vasodilation of resistance vessels and relaxation of precapillary sphincters, by reduction of tissue PO_2_ and interstitial accumulation of vasoactive metabolites, such as H^+^, K^+^, and adenosine (23). Moderate increases in tissue metabolism seem to be associated with augmented diffusive oxygen delivery, whereas greater increases in tissue metabolism seem to be supported by augmentation of convective oxygen delivery (31).

Endothelial vasoactive compounds modulate vascular resistance during changes in shear stress generated by blood flow against the static endothelium (26). The principal relaxing factor is nitric oxide (NO) (32–34). The principal constricting factor is endothelin-1 (ET-1) (35). Although activation of both ET_A_- and ET_B_-receptors on smooth muscle cells leads to vasoconstriction, activation of ET_B_-receptors on endothelial cells leads to NO-mediated vasodilation (35).

It has been suggested that the enteric nervous system participates in regulation of intestinal oxygenation, however, as this seems to be mediated via endothelial release of nitric oxide (NO), this will not be discussed separately (36). A direct effect of gastrointestinal hormones and peptides in the regulation of intestinal oxygenation has not been clearly established and will therefore not be further addressed in this review (23).

### Extrinsic Regulation of Intestinal Oxygenation

Extrinsic mechanisms include neural factors and circulating vasoactive compounds (22). The physiological role of these compounds, including norepinephrine, angiotensin II, vasopressin, histamine, and bradykinin, is uncertain, as these were mostly studied using exogenous administration (23).

Splanchnic nerve stimulation produces a pattern of changes in the intestinal vasculature that is characterized by three phases: a constrictor phase, an escape phase, and a hyperemic phase (23). First, nerve stimulation leads to constriction of resistance vessels and closure of precapillary sphincters. However, during continued sympathetic stimulation, blood flow partially recovers. It is suggested that accumulation of local metabolites and/or release of vasodilators from sensory nerves causes this autoregulatory escape by relaxation of previously constricted resistance vessels (37). Cessation of sympathetic stimulation is followed by a hyperemic phase before blood flow gradually returns to baseline (38). This post-stimulatory hyperemia may be explained by vasodilator metabolite release during the escape phase (39).

The intestine is extensively innervated by parasympathetic fibers originating from the vagus nerve. Although these fibers may have an indirect effect on intestinal oxygenation via changes in intestinal motility and secretion, there does not appear to be a direct vasoactive effect (23).

Several reviews on post-natal maturation of these intrinsic and extrinsic mechanisms describe that some may not be functionally mature at birth, whereas others may functionally decline in the post-natal period (9, 40–44). However, these reviews do not provide guidance for clinical practice, specifically with regard to preterm neonates. Therefore, our aim was to review the literature on maturation of intestinal oxygenation mechanisms and translate these changes into clinical observations with potential implications for preterm neonates.

## Methods

A literature search was conducted to evaluate reports on the post-natal maturation of intestinal oxygenation mechanisms in neonates. The search strategy is presented in Table 1. English-language articles were selected only if they included a comparison between newborn and mature intestinal oxygenation. Furthermore, as we were mostly interested in baseline intestinal hemodynamics, we excluded articles in which external influences or interventions were investigated. In addition to the database search, we screened the reference lists of all relevant articles for additional publications. To identify clinical studies in preterm neonates, the search strategy was repeated, following the same stepwise procedure, with several additional search terms, as presented in Table 1. We excluded articles that included only sick infants or studied the effect of external influences. In case a control group was included, we included the article, but only present results for the control group.

**Table 1:** Search strategy.

**^#^**	**Searches**	**Results**
**Postnatal maturation of intestinal oxygenation mechanisms**		
1	Intestine, small [mh]	160,222
2	Splanchnic circulation/physiology [mh]	2,523
3	Intestine, small/blood supply^*^ [mh]	6,624
4	Intestine, small/physiology [mh]	52,907
5	Mesenteric arteries [mh]	16,536
6	Splanchnic [tiab]	9,642
7	Abdom^*^ [tiab]	344,335
8	Intestin^*^ [tiab]	361,977
9	Mesenter^*^ [tiab]	59,868
10	^#^1 OR ^#^2 OR ^#^3 OR ^#^4 OR ^#^5 OR ^#^6 OR ^#^7 OR ^#^8 OR ^#^9	815,899
11	Hemodynamics/physiology [mh]	153,990
12	Vascular resistance/physiology [mh]	7,376
13	Vasoconstriction/physiology [mh]	6,745
14	Vasodilation/physiology [mh]	10,143
15	Hemodynamics [tiab]	48,463
16	Vascular resistance [tiab]	31,064
17	Vasoconstrict^*^ [tiab]	42,933
18	Vasodilat^*^ [tiab]	67,236
19	Perfusion [tiab]	159,441
20	Circulat^*^ [tiab]	403,596
21	Blood flow [tiab]	167,197
22	Oxygenation [tiab]	51,192
23	^#^11 OR ^#^12 OR ^#^13 OR ^#^14 OR ^#^15 OR ^#^16 OR ^#^17 OR ^#^18 OR ^#^19 OR ^#^20 OR ^#^21 OR ^#^22	924,027
24	^#^10 AND ^#^23	49,658
25	Infant, Newborn [mh]	600,550
26	Infant [tiab]	216,859
27	Newborn [tiab]	137,450
28	Neonat^*^ [tiab]	263,693
29	Post-natal [tiab]	103,008
30	Develop^*^ [tiab]	4,280,256
31	^#^25 OR ^#^26 OR ^#^27 OR ^#^28 OR ^#^29 OR ^#^30	5,003,395
32	^#^24 AND ^#^31	9,837
**Clinical observations**		
33	Ultrasonography [mh]	70,985
34	Doppler [tiab]	102,865
35	Spectroscopy, Near-Infrared [mh]	13,026
36	Near-infrared spectroscopy [tiab]	11,392
37	^#^33 OR ^#^34 OR ^#^35 OR ^#^36	490,849
38	^#^32 AND ^#^37	330
39	Preterm [tiab]	67,490
40	^#^38 AND ^#^39	145

# *search number*;

* truncation; mh, MeSH term; tiab, title or abstract

## Results

**Figure 2:**
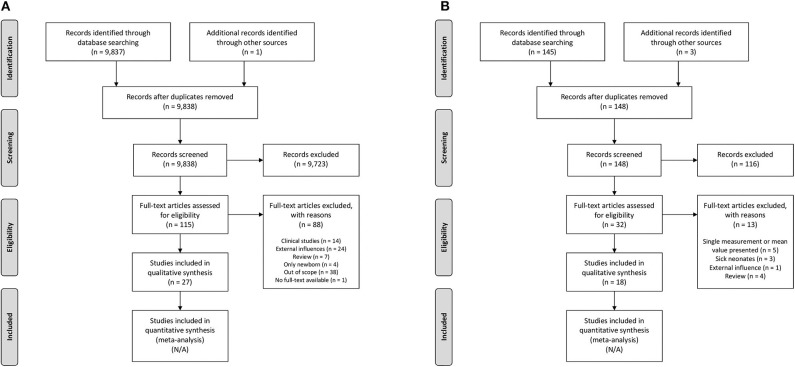
Search strategy. **(A)** Postnatal maturation of intestinal oxygenation mechanisms, **(B)** Clinical observations. N/A, not applicable.

Our initial search resulted in 9,837 articles. We assessed titles and abstracts of all articles, of which 114 appeared relevant. One additional publication was ascertained using the reference lists within these articles. After reading the full texts, 27 articles were included in this review (Figure 2A). The main findings are presented in Table 2.

**Table 2:** Postnatal maturation of intestinal oxygenation mechanisms.

**Year**	**Author**	**Species**	***n***	**Ages**	**Newborn**	**Adolescent/adult**	**Comparison**
**Intrinsic mechanisms**							
1988	Crissinger et al. (45)	Swine	20	1, 3 d, 2 wk, 1 mo	Venous pressure elevations led to reductions in blood flow and increases in (a–v)O_2_. Oxygen uptake increased in animals aged 1 day and remained unchanged in animals aged 3 days. Total vascular resistance decreased in response to venous pressure elevation in animals aged 1 day and increased in animals aged 3 days.	Venous pressure elevation led to decreased blood flow, increased (a–v)O_2_ and decreased oxygen uptake. Total vascular resistance increased in response to venous pressure elevations.	Venous pressure elevation led to greater reductions in blood flow and oxygen uptake in all older animals compared to animals at day 1. Predominance of metabolic factors at day 1 and myogenic factors in older animals is suggested as evidenced by the total vascular resistance.
1988	Nowicki et al. (46)	Swine	18	3, 35 d	Blood flow decreased in response to reductions in perfusion pressure. Oxygen uptake increased in response to reductions in perfusion pressure. Pressure-flow autoregulation was absent.	Blood flow decreased in response to reductions in perfusion pressure. Oxygen uptake increased in response to reductions in perfusion pressure. Pressure-flow autoregulation was present.	Pressure-flow autoregulation was only present in older animals. Blood flow decreased and oxygen uptake increased to a similar extent in both age groups. As a consequence, oxygen uptake was more effectively maintained in older animals.
1990	Nowicki et al. (47)	Swine	26	3, 35 d	During free flow, blood flow decreased and (a–v)O_2_ initially remained unchanged in response to venous pressure elevation, therefore oxygen uptake decreased. Greater venous pressure elevation led to increased (a–v)O_2_ and maintained oxygen uptake.	During free flow, blood flow decreased and (a–v)O_2_ remained unchanged in response to venous pressure elevation, therefore oxygen uptake decreased.	In response to venous pressure elevation, blood flow and oxygen uptake decreases to a greater extent in newborn animals.
1991	Nowicki et al. (48)	Swine	15	3, 35 d	Oxygen uptake decreased in response to arterial pressure reduction. Pressure-flow autoregulation was absent.	Oxygen uptake was maintained during arterial pressure reduction. Pressure-flow autoregulation was present and associated with venous PO_2_, but not with blood flow.	Pressure-flow autoregulation was only present in older animals. Oxygen uptake was only maintained in older animals.
1992	Nowicki et al. (49)	Swine	14	3, 35 d	Increased oxygen demand led to greater oxygen uptake resulting from increased (a–v)O_2_. Pressure-flow autoregulation was absent.	Increased oxygen demand led to greater oxygen uptake resulting from increased (a–v)O_2_. Pressure-flow autoregulation was present.	Pressure-flow autoregulation was only present in older animals.
1993	Nowicki et al. (50)	Swine	24	3, 35 d	Pressure-flow autoregulation was absent. The (a–v)O_2_ increased in response to perfusion pressure reductions, but the magnitude of the increase diminished with lower pressures. Oxygen uptake decreased significantly in response to perfusion pressure reductions.	Pressure-flow autoregulation was present. The (a–v)O_2_ increased in response to perfusion pressure reductions. Oxygen uptake only decreased in response to the greatest reduction in perfusion pressure	Pressure-flow autoregulation was only present in older animals. Oxygen uptake was only maintained in older animals.
1995	Nankervis et al. (51)	Swine	10	3, 35 d	Inhibited NO production increased vascular resistance and (a–v)O_2_, no changes in oxygen uptake were observed. Vasodilation was observed in response to increased flow. Inhibited NO production increased vascular resistance during increased flow rates.	Inhibited NO production did not affect vascular resistance, nor oxygen uptake. Vasodilation was observed in response to increased flow. Inhibited NO production did not have an effect on vascular resistance during increased flow.	Only in newborn animals, the NO-cGMP axis participates in setting basal vascular resistance and in flow-induced dilation. Flow-induced dilation was present in both age groups.
1997	Nakanishi et al. (52)	Rabbits	36	3–5 d, 4–8 mo	Inhibited Ca^2+^-influx caused vasorelaxation. Stimulated Ca^2+^-release from intracellular store sites caused vasoconstriction.	Inhibited Ca^2+^-influx caused vasorelaxation. Stimulated Ca^2+^-release from intracellular store sites caused vasoconstriction.	Newborn animals showed greater vasoconstriction in response to stimulated Ca^2+^-release from intracellular stores, whereas older animals showed greater vasorelaxation in response to inhibited Ca^2+^-influx through Ca^2+^-channels across the sarcolemma.
1998	Nowicki (53)	Swine	14	3, 35 d	Blood flow decreased, (a–v)O_2_ increased and oxygen uptake decreased in response to reduced flow, achieved by perfusion pressure reductions. Vascular resistance increased in response to perfusion pressure reductions.	Blood flow decreased, (a–v)O_2_ increased and oxygen uptake remained unchanged in response to reduced flow, achieved by perfusion pressure reductions. Vascular resistance increased in response to perfusion pressure reductions.	In newborn animals, vascular resistance increases to a greater extent in response to perfusion pressure reductions. Oxygen uptake is only maintained in older animals during perfusion pressure reductions.
1998	Reber et al. (54)	Swine	14	3, 35 d	Myogenic vasoconstriction in response to increased intravascular pressure was present. Flow-mediated dilation was present. Vasodilation was noted in response to combined increases in pressure and flow. Pressure-flow autoregulation was absent.	Myogenic vasoconstriction in response to increased intravascular pressure was absent. Flow-mediated dilation in response to increased flow was present. A modest degree of pressure-flow autoregulation was observed.	Myogenic vasoconstriction was only observed in newborn animals. A greater degree of flow-induced dilation was observed in newborn animals. Vasodilation in response to combined increases in pressure and flow was only observed in newborn animals. Pressure-flow autoregulation was only present in older animals.
1999	Nowicki (55)	Swine	50	3, 35 d	Vasoconstriction was observed in response to reduction of flow rate. Inhibited NO production increased vascular resistance. Low flow conditions caused increased vasoconstriction in response to ET-1 and these effect were even greater during inhibited NO production.	Vasoconstriction was observed in response to reduction of flow rate. Inhibited NO production increased vascular resistance. Low flow conditions did not alter the response to ET-1.	In newborn animals, low flow conditions caused greater vasoconstriction and a greater vasoconstrictor response to ET-1. Inhibited NO production increased vascular resistance to a greater extent in newborn animals.
2000	Nankervis et al. (56)	Swine	10	3, 35 d	Infusion of ET-1 caused vasoconstriction and decreased (a–v)O_2_. Oxygen uptake was compromised during ET-1 infusion. Blockade of ET_A_-receptors did not alter basal vascular tone. Blockade of ET_B_-receptors increased the extent of vasoconstriction, but had no effect on (a–v)O_2_.	Infusion of ET-1 caused vasoconstriction and decreased (a–v)O_2_. Oxygen uptake was compromised during ET-1 infusion. Blockade of ET_A_-receptors did not alter basal vascular tone. Blockade of ET_B_-receptors had no effect on vessel diameter, nor (a–v)O_2_.	In newborn animals, endogenous ET-1 participates in exchange vessel regulation, but not in setting basal vascular tone. Vasoconstriction caused by endogenous ET-1 is offset by vasodilation by activation of ET_B_-receptors, but only in newborn animals. In newborn animals, ET-1 infusion leads to greater increases in (a–v)O_2_.
2000	Nankervis et al. (57)	Swine	10	3, 35 d	Blockade of ET_A_-receptors did not alter basal vascular resistance, but increased oxygen uptake. Blockade of ET_B_-receptors and removal of the endothelium increased vasoconstriction in response to ET-1.	Blockade of ET_A_-receptors did not alter basal vascular resistance, nor oxygen uptake. Blockade of ET_B_-receptors and removal of the endothelium did not alter vasoconstriction in response to ET-1.	ET_B_-receptors are located on the endothelium and modulate the vasoconstrictor response to ET-1, but only in newborn intestine.
2001	Nankervis et al. (58)	Swine	10	1, 40 d	Myogenic vasoconstriction was observed in response to increased vascular pressure under no-flow circumstances, whereas vasodilation was observed in the presence of flow. Blockade of ET_A_-receptors caused vasodilation, but only in the absence of flow. Blockade of ET_B_-receptors and NO production caused vasoconstriction regardless of flow conditions.	Myogenic vasoconstriction in response to increased arterial pressure was absent, instead vasodilation was observed. Blockade of ET_A_-receptors, ET_B_-receptors and NO production produced no effect on vessel diameter.	Myogenic vasoconstriction in response to increased arterial pressure was only observed in newborn animals. In newborn animals, ET-1 participates in setting basal vascular tone, independent of the myogenic mechanism, that is offset by activation of ET_B_-receptors.
2001	Nankervis et al. (59)	Swine	12	1, 40 d	ET_A_-receptors were present and localized to vascular smooth muscle. ET_B_-receptors were present and localized to the endothelium.	ET_A_-receptors were present and localized to vascular smooth muscle. ET_B_-receptors were present and localized to the endothelium.	ET_A_- and ET_B_-receptors are present in a greater quantity in newborn intestine compared with mature intestine.
2001	Reber et al. (60)	Swine	10	3, 35 d	NO production increases and vascular resistance decreasing in response to increases in flow rate. Increased NO production was observed in response to decreases in flow rate, but vascular resistance remained unchanged.	NO production and vascular resistance remained unaltered in response to both increases and decreases in flow rate.	Basal NO production and stimulated NO production in response to increased flow rate were greater in newborn animals. Only in newborn animals, flow-induced dilation was observed.
2002	Reber et al. (61)	Swine	30	1, 3, 10, 30 d	Expression of eNOS protein was present. Blockade of NO production increased vascular resistance.	Expression of eNOS protein was present. Blockade of NO production increased vascular resistance at day 10, but not at day 30.	Expression of eNOS protein increased until day 10, but then decreased until day 30, whereas eNOS mRNA remained stable. Compared to 1-day-old animals, vascular resistance was higher in 30-day-old animals. Oxygen uptake increased until day 3, but then decreased until day 30.
2003	Su et al. (62)	Swine	40	1, 10 d	Vasoconstriction was observed in response to increased intravascular pressure. Blockade of PKC eliminated this vasoconstrictor response. Activation of PKC increased the contractile response.	No change in vessel diameter was observed in response to increased intravascular pressure. Neither blockade of PKC nor activation of PKC produced any changes in vessel diameter.	The intensity of myogenic vasoconstriction is greater in newborn animals. Myogenic vasoconstriction was attenuated by blockade and activation of PKC, but only in newborn animals.
2004	Su et al. (63)	Swine	12	3, 10, 30 d	ET_A_- and ET_B_-receptor mRNA and protein expression was present. The ET_A_-receptor was localized to vascular smooth muscle and the ET_B_-receptor was localized to the endothelial layer.	ET_A_- and ET_B_-receptor mRNA and protein expression was present. The ET_A_-receptor was localized to vascular smooth muscle and the ET_B_-receptor was localized to the endothelial layer.	ET_A_- and ET_B_-receptor mRNA and protein expression was greater in newborn animals. Localization of ET_A_- and ET_B_-receptors was similar.
2005	Wendel et al. (64)	Rats	15	0, 5, 14, 21, 28 d, adult	ET_B_-receptors were absent on smooth muscle cells in the mesenteric circulation	From day 14, ET_B_-receptors were present on smooth muscle cells of mesenteric arterioles, but not arteries and veins	ET_B_-receptors are only present on smooth muscles cells of mesenteric arterioles in mature intestine
2020	Ayuso et al. (65)	Swine	22	0, 3, 8, 19 d	In LBW animals, eNOS is present at birth in a moderate degree. In NBW animals, eNOS expression peaks at birth.	In both LBW and NBW, animals, eNOS expression is present in a moderate degree.	eNOS expression is greater in newborn animals than in mature animals, but only in NBW animals.
**Extrinsic mechanisms**							
1985	Buckley et al. (66)	Swine	34	1, 2–4 d, 1, 2 wk, 1 mo	An increase in vascular resistance was observed in response to inhibition of the baroreceptor reflex, achieved by occlusion of the carotid arteries. The circulation is under neural vasoconstrictor tone, as evidenced by decreased vascular resistance in response to section of the splanchnic nerve. SpNS produced vasoconstriction.	An increase in vascular resistance was observed in response to inhibition of the baroreceptor reflex, achieved by occlusion of the carotid arteries. The circulation is under neural vasoconstrictor tone, as evidenced by decreased vascular resistance in response to section of the splanchnic nerve. Increased vascular resistance was observed in response to SpNS and MNS.	The mesenteric circulation participated in the baroreceptor reflex in all age groups. Neural factors participate in setting basal vascular tone from birth onwards, but the decrease in vascular resistance observed in response to splanchnic nerve section was greater in older animals. Vascular resistance increased to a greater extent in older animals in response to SpNS.
1987	Buckley et al. (67)	Swine	34	6 h-2 d, 4–7 d, 2 wk, 1, 2 mo	Inhibition of the baroreceptor reflex increased vascular resistance. Severing the major components of the innervation increased flow. Vasoconstriction was observed in response to mesenteric nerve stimulation. Autoregulatory escape in response to sustained MNS was not observed.	Inhibition of the baroreceptor reflex increased vascular resistance. Severing the major components of the innervation increased flow. Vasoconstriction was observed in response to mesenteric nerve stimulation. Autoregulatory escape in response to sustained MNS was observed.	From birth, the mesenteric circulation is under neural vasoconstrictor tone and participates in the baroreceptor reflex. The increase in vascular resistance was greater and the latencies for the onset of vasoconstriction in response to MNS were smaller in older animals. From the age of 2 weeks, autoregulatory escape during sustained MNS is demonstrable and it is well-established by the end of the first month.
1991	Nowicki et al. (68)	Swine	22	3, 35 d	Vasoconstriction was observed in response to MNS. Autoregulatory escape was observed in response to sustained MNS, however oxygen uptake remained below baseline.	Vasoconstriction was observed in response to MNS. Autoregulatory escape was observed in response to sustained MNS, however oxygen uptake remained below baseline.	Sustained MNS produced similar effects on vascular resistance and oxygen uptake in newborn and older animals. Both age groups demonstrated autoregulatory escape.
1996	Hoang et al. (69)	Swine	22	0–2, 10–14 d	Neither α_1_- and α_2_-adrenoceptors seem to play a role in the vasoconstrictor response to α_1_- and α_2_-agonists, as evidenced by an unaltered response in the presence of α_1_- and α_2_-antagonists.	Specific, functional α_1_- and α_2_-adrenoceptors were present, as evidenced by blockade of the vasoconstrtictor response to α_1_- and α_2_-agonists in the presence of α_1_- and α_2_-antagonists, respectively.	Selectivity of α_1_- and α_2_-adrenoceptor activity was only observed in older animals.
1998	Nowicki (70)	Swine	10	3, 35 d	SP is present. Infusion of SP causes vasodilation and increases oxygen uptake. Blockade of SP NK-1 receptors increases basal vascular resistance. Blockade of NO production eliminates SP-induced vasodilation and increases basal vascular resistance.	SP is present. Infusion of SP causes vasodilation and increases oxygen uptake. Blockade of SP NK-1 receptors did not alter basal vascular resistance. Blockade of NO production eliminates SP-induced vasodilation.	SP content is greater in newborn animals. SP participates in setting basal vascular resistance, but only in newborn animals.
2007	Gonzáles-Luis et al. (71)	Swine	24	1, 2 wk	Electrical field stimulation, in the absence of cholinergic and adrenergic components, produced vasodilation, that was eliminated during blockade of NO production.	Electrical field stimulation, in the absence of cholinergic and adrenergic components, produced vasodilation.	Non-adrenergic, non-cholinergic relaxation was greater in newborn animals and was eliminated during blockade of NO production.

*(a–v)O_2_: arteriovenous oxygen content difference, d, days, eNOS, endothelial isoform of NO synthase; ET, endothelin; LBW, low birth weight; MNS, mesenteric nerve stimulation; mo, month; NBW, normal birth weight; NO, nitric oxide; PKC, protein kinase C; PO2, partial pressure of oxygen; SP, Substance P; SpNS, splanchnic nerve stimulation; wk, week*.

### Postnatal Maturation of Mechanisms Regulating Intestinal Oxygenation

In the neonatal intestine, basal vascular resistance seems to be determined by myogenic factors, endothelial vasoactive compounds, and neural factors, based on observations in newborn swine (45, 51, 55, 58, 61, 66, 67). Using a swine model, Nowicki et al. (46) demonstrated in several studies that pressure-flow autoregulation is absent in neonatal intestine (54). Therefore, decreases in arterial pressure result in decreased intestinal blood flow. Moreover, these studies indicate that subsequent increases in oxygen extraction are insufficient to meet intestinal oxygen demand (48, 50, 53). The absence of pressure-flow autoregulation may indicate immaturity of the myogenic and/or metabolic mechanisms described above. However, myogenic vasoconstriction has been observed in newborn swine in response to increases in venous pressure (45, 47, 54, 58, 62). Therefore, it seems more likely that the absence of pressure-flow autoregulation reflects immaturity of the metabolic mechanism. Neonatal intestine may thus lack an important vasodilator mechanism.

Moreover, there may be a greater amount of constricting factors, as demonstrated by a greater quantity of ET_A_- and ET_B_-receptors in newborn swine compared to mature swine (59, 63). In the presence of these vasoconstrictor influences, i.e., the myogenic mechanism and ET-1, there seems to be an important role for NO as a vasodilator to maintain intestinal oxygenation in neonates. This assumption is supported by findings of Reber et al. (60, 61) who demonstrated that NO production is considerably greater in newborn swine compared with mature swine under both basal and stimulated conditions.

In mature intestine, basal vascular resistance seems to be determined by passive-elastic characteristics of the vasculature rather than active constrictor or dilator tone, based on observations in mature swine (42, 46, 53). Pressure-flow autoregulation is present in mature swine and seems to be associated with venous PO_2_, consistent with the metabolic mechanism described above (46, 48–50, 53, 54). Therefore, during decreases in arterial pressure, blood flow is still maintained in mature swine compared to newborn swine and concomitant increases in oxygen extraction may enable mature intestine to more consistently meet oxygen demand (46, 48–50, 53, 54). In contrast to newborn swine, myogenic vasoconstriction in response to venous pressure elevation seems to be absent in mature swine (47, 54, 58, 62). As the influence of vasoconstrictor forces, i.e., the myogenic mechanism and ET-1, decreases with advancing post-natal age, it follows that mature intestine may not require the same vasodilator forces as neonatal intestine. Indeed, several studies showed that NO production and the degree of flow-induced vasodilation are considerably smaller in mature swine than in newborn swine (51, 54, 60, 61).

In conclusion, during post-natal maturation, the contribution of the metabolic mechanism in regulation of intestinal oxygenation increases, whereas the influences of the myogenic mechanism and endothelial vasoactive compounds decrease. In neonatal intestine, pressure-flow autoregulation is not yet functional, possibly due to immaturity of the metabolic mechanism.

### Translation to Clinical Observations

To the best of our knowledge, there are no studies available that investigate post-natal maturation of intestinal oxygenation mechanisms in preterm intestine, nor studies that compared maturation of intestinal oxygenation mechanisms between preterm and term intestine.

Therefore, we used the clinical studies that were identified by the additional search strategy to translate the results into clinical observations for preterm neonates. Our additional search resulted in 145 articles. We assessed titles and abstracts of all articles, of which 29 appeared relevant. Three additional publication were ascertained using the reference lists within these articles. After reading the full texts, 18 articles were included in this review (Figure 2B). The main findings are presented in Table 3.

**Table 3:** Clinical studies in preterm neonates.

**Year**	**Author**	**Population**	***n***	**Measurements**	**Postnatal changes**
**Doppler**					
1990	Van Bel et al. (16)	Preterm and term (GA 24–43 wk)	91	1–5, daily	PI increased between 1 and 6 days. No significant changes were observed for PSV, TAMV, and EDV. Values for PSV, TAMV, and EDV increased with advancing GA. PI was not associated with GA. In SGA infants, EDV was significantly higher on day 1 and PI was significantly lower on days 1–2 compared to AGA infants.
1992	Coombs et al. (72)	Preterm (GA 27–35 wk) and term (GA 37–41 wk)	18	1, 2, 4 d	In term infants, an increase in PSV was observed between 1 and 2 days. In preterm infants, no significant changes were observed in PSV between 1 and 4 days. No significant difference was observed for PSVbetween term and preterm infants.
1996	Martinussen et al. (73)	Preterm (GA 33–35 wk)	15	1–7 d, daily	An increase in TAMV and EDV was observed between 1 and 2 days. EDV was positive in all infants examined between 6 and 24 h. No changes were observed after day 3.
1999	Maruyama et al. (74)	Preterm (GA 28–33 wk)	44	1–6 d, daily	An increase in TAMV was observed between 1 and 6 days. A decrease in RI was observed between 1 and 2 days, and then an increase to 6 days. A decrease in RVR was observed between 1 and 6 days.
1999	Yanowitz et al. (75)	VLBW (BW 750–1,250 g)	20	6, 30, 54 h, 7, and 14 d	An increase in TAMV was observed between 6 h and 7 days and between 6 h and 14 days. No significant changes were observed in EDV and RVR.
2001	Maruyama et al. (76)	VLBW, SGA (BW <1,500 g and below the 10th percentile	10	1–7 d, daily	An increase in PSV was observed between 1 and 6–7 days. An increase in TAMV was observed between 1 and 5–6 days. An increase in EDV was observed between 1 and 3–7 days. A decrease in RI was observed between 1 and 2–7 days. A decrease in RVR was observed between 1–6 days. PSV, TAMV, and EDV were lower in SGA infants compared to AGA infants.
2006	Havranek et al. (77)	Preterm (GA <34 wk)	25	1–5 d, daily	An increase in PSV and TAMV was observed between 1 and 5 days.
2009	Papacci et al. (78)	Preterm (GA 25–28 wk, 29–32 wk, 33–36 wk) and term (GA 37–41 wk)	69	1, 3, 7, 14, 21, and 28 d	An increase in PSV, EDV and TAMV was observed between 1 and 28 days. Values for PSV, EDV, and TAMV increased with advancing GA. No changes in PI an RI were observed.
2012	Havranek et al. (79)	VLBW (BW <1,500 g)	35	1, 3, 5, 7, 10, and 14 d	An increase in PSV and TAMV was observed between 1 and 14 days. Lower values on day 1 were associated with higher post-natal increases in PSV and TAMV. No change in EDV was observed. No correlation was found between GA and day 1 PSV and TAMV.
2014	Thompson et al. (80)	Preterm (GA <27 wk, 27–31 wk, and 31–36 wk)	41	1, 1–4, 5–7, 8–14, and 15–28	No differences were observed in PSV on day 1 between GA groups. However, on day 5–7 and 8–14 higher GA was associated with higher PSV.
2015	Gursoy et al. (81)	Preterm (GA 26–34 wk)	25	1–5 d, daily	An increase in PSV, TAMV and EDV was observed between 1 and 5 days. A decrease in RI was observed between 1 and 5 days.
2018	Kocvarova et al. (82)	Preterm (GA 34–37 wk) and term (GA 38–42 wk)	40	2, 24, and 72 h	An increase in PSV was observed between 2 and 72 h. An increase in EDV was observed between 2 and 24 h. All EDV values were positive at 24 h. An increase in TAMV was observed between 2 and 24 h and between 24 and 72 h. A decrease in PI and RI was observed between 2 and 24 h. Preterm infants had lower PI at 2 h and higher PSV and EDV at 24 h compared to term infants.
**NIRS**					
2010	Cortez et al. (83)	Preterm (GA ≤ 30 wk)	19	48 h-14 d, continuously	A decrease in daily mean r_s_SO_2_ was observed between 48 h until 9 days, and then an increase between 10 and 14 days.
2011	McNeill et al. (84)	Preterm (GA 29–30 wk and 32–33 wk)	12	0–21 days, continuously	A decrease in daily mean r_s_SO_2_ was observed between 1 and 7 days in infants with GA 29–30 wk and between 1 and 4.5 days in infants with GA 32–33 wk. Afterwards, an increase in r_s_SO_2_ was observed. Lower GA was associated with lower r_s_SO_2_ values.
2014	Patel et al. (85)	Preterm (GA <32 wk and birth weight <1,500 g)	92	0–7 d, 5 min daily	An increase in mean r_s_SO_2_ was observed between 1 and 3 days. Afterwards, a decrease in r_s_SO_2_ was observed.
2016	Bozzetti et al. (86)	Preterm (GA 29–33 wk)	20	0–24, 48–72, 3 h daily	A decrease in r_s_SO_2_ was observed between 0–24 h and 48–72 h. In IUGR infants r_s_SO_2_ was significantly lower compared to non-IUGR infants.
2017	Ledo et al. (87)	Preterm (GA <32 wk)	72	36 h-7 d, continuously	An initial decrease in r_s_SO_2_ was observed for all infants, regardless of DA status. An increase in r_s_SO_2_ was observed after closure of the DA at day 3. No increase in r_s_SO_2_ until day 7 was observed in infants with persistent hsPDA.
2019	Kuik et al. (88)	Preterm (GA <30 wk, or birth weight <1,000 g, or GA <32 wk and birth weight <1,200 g	29	2–5, 8, 15, 22, 29, 36 d, 2 h on each day	Generally, an increase in r_s_SO_2_ was observed between 2 and 36 days, with the lowest values on day 4 and day 15.

*BW, birth weight; d, days; DA, ductus arteriosus; EDV, end-diastolic velocity; GA, gestational age; h, hours; NIRS, near-infrared spectroscopy; PI, pulsatility index; PSV, peak systolic velocity; RI, resistance index; r_s_SO_2_, splanchnic oxygen saturation; RVR, relative vascular resistance; TAMV, time-averaged mean velocity; VLBW, very low birth weight; wk, weeks*.

In preterm neonates, intestinal perfusion increases in early life, as demonstrated by increases in peak systolic flow (PSV) and time-averaged mean velocity (TAMV), measured with Doppler, until day 28 of life (73–79, 81). Despite increases in blood flow during the first weeks of life, splanchnic oxygen saturation (r_s_SO_2_), estimated using near-infrared spectroscopy (NIRS), initially decreases in the first week of life and then increases until day 21 after birth (83, 84, 86–88). Advancing gestational age (GA) is associated with both higher blood flow and higher r_s_SO_2_ (78, 80, 84).

## Interpretation and Discussion

Our review of the literature shows that pressure-flow autoregulation is only present in mature intestine, as demonstrated in animal studies. In contrast, neonatal intestine relies on increases in oxygen extraction to meet oxygen demand during decreases in arterial pressure. Clinical observations demonstrate lower baseline hemodynamic and oxygenation characteristics in preterm compared to term neonates. These results suggest a developmental delay of vasodilator forces and a smaller reserve to increase intestinal oxygen extraction in preterm neonates that may endanger intestinal oxygenation during decreases in arterial pressure.

Oxygen requirements of neonatal intestine, and specifically those of preterm neonatal intestine, may be substantial, due to a high nutritional demand to support growth and maturation processes (7). Our review of the literature shows that preterm neonates may not be able to meet these requirements, as pressure-flow autoregulation is still absent and increases in oxygen extraction are insufficient to meet tissue oxygen demand (46, 48–50, 53, 54). Clinical studies showed that r_s_SO_2_ initially decreases after birth, possibly indicating that oxygen extraction is already maximized under basal conditions (83, 84, 86, 87). Although the course of intestinal blood flow and r_s_SO_2_ in early post-natal life has not been studied with simultaneous Doppler and NIRS measurements, the initial decrease in r_s_SO_2_ suggests that the increase in oxygen extraction is greater than the increase in blood flow during the first days of life. We hypothesize that this could be due to patency of the ductus arteriosus. This hypothesis is supported by findings of Ledo et al. (87) who investigated the effect of ductal patency on the course of r_s_SO_2_ in preterm neonates and found that the increases in r_s_SO_2_ from day 3 of life are paralleled by ductal closure. The initial decrease in r_s_SO_2_ may thus be explained by ductal steal, resulting in reduced diastolic flow in the descending aorta, resulting in a decreased intestinal perfusion pressure, and in the absence of intestinal pressure-flow regulation, preterm intestine relies on increases in oxygen extraction (73, 74, 80, 82, 87). This may enable preterm neonates to meet intestinal oxygen requirements during baseline conditions, yet creates unfavorable conditions during periods of additional stress. A recent review by Chaaban et al. (44) showed that the predominant response of neonatal intestine to decreases in oxygen delivery or increases in oxygen demand is increased oxygen extraction. In preterm neonates, however, it may not be possible to further increase oxygen extraction. Therefore, during periods of additional oxygen requirements, preterm neonates may fail to meet intestinal oxygen requirements, leading to disruption of the intestinal barrier and reduced nutrient absorption.

There may be an important role for NO to facilitate oxygen delivery in neonatal intestine during baseline conditions. Our review of the literature demonstrates that NO counteracts active vasoconstrictor tone, induced by myogenic factors and ET-1. Clinical studies showed that intestinal blood flow increases with advancing post-natal age, possibly indicating maturation of vasodilator forces. Although no causal relation between intestinal blood flow and NO production has been demonstrated, Reber et al. (60, 61) found that the post-natal increase in intestinal blood flow is paralleled by increases in NO production in neonatal swine. Reber et al. (9) hypothesized that loss of NO production may compromise intestinal oxygen delivery and thus contribute to intestinal injury in neonates. This hypothesis is supported by findings of Nowicki et al. (89) who showed that NO-mediated vasodilation was disrupted in human intestine resected for NEC. The pathophysiology of NEC is complex and has not been fully elucidated, but may include impaired intestinal microcirculation (14). In preterm neonates, loss of NO production may thus predispose the intestine to hypoxic tissue injury, possibly contributing to the development of NEC.

We acknowledge some limitations. First, our review describes the mechanistic strategies available to neonatal and mature intestine to maintain adequate oxygenation, but does not include the contribution of these strategies during external influences that may alter intestinal oxygen supply or demand, e.g., anemia and enteral feeding. Nevertheless, our speculations on implications of these external influences on intestinal oxygenation are supported by recent reviews in both animal models and preterm neonates (18, 19, 44). Second, we purposely did not take into account factors that may influence the maturation processes described. These factors may include structural maturation and growth of intestinal tissue and vascularization networks, microbial colonization, and increasing volumes of enteral feeding (6, 90–92). Third, inherent limitations in Doppler and NIRS techniques complicate the translation to clinical implications. Doppler requires trained personnel, is prone to operator-dependent bias and provides only momentary blood flow velocity measurements of large vessels, whereas NIRS is challenged by intraindividual variability and interference of other tissues, intestinal contents and bowel movements with splanchnic oxygen saturation measurements (18). Finally, translation of our findings, derived from animal studies, to implications for preterm neonates is complicated by interspecies differences. By using NIRS to more regularly monitor r_s_SO_2_ in preterm neonates, we may be able to learn more about the maturational phenomena described previously. For instance, it may provide insight in the influence of GA and post-natal age on basal intestinal oxygenation and it may be used to study the response to external influences that alter oxygen supply or oxygen demand. At the same time, a better understanding of post-natal maturation of intestinal oxygenation mechanisms may facilitate interpretation of r_s_SO_2_ and advance the use of NIRS in a clinical setting. Ultimately, bedside r_s_SO_2_-monitoring may lead to more streamlined and personalized care practices for at-risk neonates.

In conclusion, preterm intestine may have a smaller reserve for perturbations in intestinal oxygen delivery and oxygen demand, as oxygen extraction may be already be maximized under baseline circumstances. Developing additional understanding on this delicate balance of oxygen supply and demand may help in guiding clinical management to prevent intestinal tissue hypoxia.

## Author Contributions

BD and EK conceptualized and designed the study. BD screened databases for eligible studies, drafted the initial manuscript, and revised the manuscript after feedback from coauthors. JMi, JMo, JH, AB, and EK critically reviewed the article. All authors contributed to the article and approved the submitted version.

## Conflict of Interest

The authors declare that the research was conducted in the absence of any commercial or financial relationships that could be construed as a potential conflict of interest.

## References

[1] Greenwood-Van Meerveld B Johnson AC Grundy D Gastrointestinal physiology and function. In: Greenwood-Van Meerveld B, editor. Gastrointestinal Pharmacology Handbook of Experimental Pharmacology. Vol. 239 Cham: Springer (2017). p. 1–16. 10.1007/164_2016_118 28176047

[2] Volk N Lacy B. Anatomy and physiology of the small bowel. Gastrointest Endosc Clin N Am. (2017) 27:1–13. 10.1016/j.giec.2016.08.001 27908510

[3] Santaolalla R Abreu MT. Innate immunity in the small intestine. Curr Opin Gastroenterol. (2012) 28:124–9. 10.1097/MOG.0b013e3283506559 22241076 PMC3502878

[4] Buettner M Lochner M. Development and function of secondary and tertiary lymphoid organs in the small intestine and the colon. Front Immunol. (2016) 7:342. 10.3389/fimmu.2016.00342 27656182 PMC5011757

[5] Lister G Walter TK Versmold HT Dallman PR Rudolph AM. Oxygen delivery in lambs: cardiovascular and hematologic development. Am J Physiol. (1979) 237:H668–75. 10.1152/ajpheart.1979.237.6.H668 517666

[6] Edelstone DI Holzman IR. Oxygen consumption by the gastrointestinal tract and liver in conscious newborn lambs. Am J Physiol. (1981) 240:G297–304. 10.1152/ajpgi.1981.240.4.G297 7223894

[7] Morgan JA Young L McCormick FM McGuire W. Promoting growth for preterm infants following hospital discharge. Arch Dis Child Fetal Neonatal Ed. (2012) 97:295–8. 10.1136/adc.2009.170910 21406452

[8] Matheson PJ Wilson MA Garrison RN. Regulation of intestinal blood flow. J Surg Res. (2000) 93:182–96. 10.1006/jsre.2000.5862 10945962

[9] Reber KM Nankervis CA Nowicki PT. Newborn intestinal circulation. Physiology and pathophysiology. Clin Perinatol. (2002) 29:23–39. 10.1016/S0095-5108(03)00063-0 11917738

[10] Widdowson EM. Development of the digestive system: Comparative animal studies. Am J Clin Nutr. (1985) 41(Suppl. 2):384–90. 10.1093/ajcn/41.2.384 3881925

[11] Dasgupta S Arya S Choudhary S Jain SK. Amniotic fluid: source of trophic factors for the developing intestine. World J Gastrointest Pathophysiol. (2016) 7:38–47. 10.4291/wjgp.v7.i1.38 26909227 PMC4753188

[12] Rhee CJ Fraser CD Kibler K Easley RB Andropoulos DB Czosnyka M. The ontogeny of cerebrovascular pressure autoregulation in premature infants. J Perinatol. (2014) 34:926–31. 10.1038/jp.2014.122 25010225 PMC4383263

[13] Nowicki PT. Ischemia and necrotizing enterocolitis: where, when, and how. Semin Pediatr Surg. (2005) 14:152–8. 10.1053/j.sempedsurg.2005.05.003 16084402

[14] Niño DF Sodhi CP Hackam DJ. Necrotizing enterocolitis: new insights into pathogenesis and mechanisms. Nat Rev Gastroenterol Hepatol. (2016) 13:590–600. 10.1038/nrgastro.2016.119 27534694 PMC5124124

[15] Clark RH Thomas P Peabody J. Extrauterine growth restriction remains a serious problem in prematurely born neonates. Pediatrics. (2003) 111(5 Pt 1):986–90. 10.1542/peds.111.5.986 12728076

[16] Van Bel F Van Zwieten PH Guit GL Schipper J. Superior mesenteric artery blood flow velocity and estimated volume flow: duplex doppler US study of preterm and term neonates. Radiology. (1990) 174:165–9. 10.1148/radiology.174.1.2403678 2403678

[17] Mintzer JP Moore JE. Regional tissue oxygenation monitoring in the neonatal intensive care unit: evidence for clinical strategies and future directions. Pediatr Res. (2019) 86:296–304. 10.1038/s41390-019-0466-9 31247635

[18] Martini S Corvaglia L. Splanchnic NIRS monitoring in neonatal care: rationale, current applications and future perspectives. J Perinatol. (2018) 38:431–43. 10.1038/s41372-018-0075-1 29472709

[19] Seager E Longley C Aladangady N Banerjee J. Measurement of gut oxygenation in the neonatal population using near-infrared spectroscopy: a clinical tool? Arch Dis Child Fetal Neonatal Ed. (2020) 105:76–86. 10.1136/archdischild-2018-316750 31154420

[20] Granger HJ Nyhof RA. Dynamics of intestinal oxygenation: interactions between oxygen supply and uptake. Am J Physiol. (1982) 243:G91–6. 10.1152/ajpgi.1982.243.2.G91 7051852

[21] Shepherd AP. Local control of intestinal oxygenation and blood flow. Annu Rev Physiol. (1982) 44:13–27. 10.1146/annurev.ph.44.030182.000305 7041790

[22] Mortillaro NA Microcirculation of the small intestine. In: Mortillaro NA, editor. The Physiology and Pharmacology of the Microcirculation. Vol. 2 Orlando, FL: Academic Press (1984). p. 57–72. 10.1016/B978-0-12-508302-7.50011-4

[23] Granger DN Richardson PD Kvietys PR Mortillaro NA Intestinal blood flow. Gastroenterology. (1980) 78:837–63. 10.1016/0016-5085(80)90692-7 6101568

[24] Stark ME Szurszewski JH. Role of nitric oxide in gastrointestinal and hepatic function and disease. Gastroenterology. (1992) 103:1928–49. 10.1016/0016-5085(92)91454-C 1333429

[25] Shepherd AP Riedel GL. Effect of pulsatile pressure and metabolic rate on intestinal autoregulation. Am J Physiol. (1982) 242:H769–75. 10.1152/ajpheart.1982.242.5.H769 7081447

[26] Pohl U Herlan K Huang A Bassenge E. EDRF-mediated shear-induced dilation opposes myogenic vasoconstriction in small rabbit arteries. Am J Physiol. (1991) 261(6 Pt 2):H2016–23. 10.1152/ajpheart.1991.261.6.H2016 1721502

[27] Bayliss WM. On the local reactions of the arterial wall to changes of internal pressure. J Physiol. (1902) 28:220–31. 10.1113/jphysiol.1902.sp000911 16992618 PMC1540533

[28] Shepherd AP. Myogenic responses of intestinal resistance and exchange vessels. Am J Physiol. (1977) 233:H547–54. 10.1152/ajpheart.1977.233.5.H547 920818

[29] Meininger GA Davis MJ. Cellular mechanisms involved in the vascular myogenic response. Am J Physiol. (1992) 263(3 Pt 2):H647–59. 10.1152/ajpheart.1992.263.3.H647 1415587

[30] Ringvold HC Khalil RA. Protein kinase C as regulator of vascular smooth muscle function and potential target in vascular disorders. Adv Pharmacol. (2017) 78:203–301. 10.1016/bs.apha.2016.06.002 28212798 PMC5319769

[31] Granger HJ Norris CP. Intrinsic regulation of intestinal oxygenation in the anesthetized dog. Am J Physiol. (1980) 238:836–43. 10.1152/ajpheart.1980.238.6.H836 7386643

[32] Ignarro LJ Buga GM Wood KS Byrns RE Chaudhurit G. Endothelium-derived relaxing factor produced and released from artery and vein is nitric oxide. Proc Natl Acad Sci USA. (1987) 84:9265–9. 10.1073/pnas.84.24.9265 2827174 PMC299734

[33] Palmer RM Ferrige AG Moncada S. Nitric oxide release accounts for the biological activity of endothelium-derived relaxing ractor. Nature. (1987) 327:524–6. 10.1038/327524a0 3495737

[34] Palmer RM Rees DD Ashton DS Moncada S. L-arginine is the physiological precursor for the formation of nitric oxide in endothelium-dependent relaxation. Biochem Biophys Res Commun. (1988) 153:1251–6. 10.1016/S0006-291X(88)81362-7 3390182

[35] Masaki T. Possible role of endothelin in endothelial regulation of vascular tone. Annu Rev Pharmacol Toxicol. (1995) 35:235–55. 10.1146/annurev.pa.35.040195.001315 7598493

[36] Surprenant A. Control of the gastrointestinal tract by enteric neurons. Annu Rev Physiol. (1994) 56:117–40. 10.1146/annurev.ph.56.030194.001001 8010737

[37] Greenway C V Scott GD Zink J. Sites of autoregulatory escape of blood flow in the mesenteric vascular bed. J Physiol. (1976) 259:1–12. 10.1113/jphysiol.1976.sp011451 957204 PMC1309011

[38] Folkow B Lewis DH Lundgren O Mellander S Wallentin I. The effect of graded vasoconstrictor fibre stimulation on the intestinal resistance and capacitance vessels. Acta Physiol Scand. (1964) 61:445–57. 14209260

[39] Remak G Hottenstein OD Jacobson ED. Sensory nerves mediate neurogenic escape in rat gut. Am J Physiol. (1990) 258(3 Pt 2):778–86. 10.1152/ajpheart.1990.258.3.H778 2316694

[40] Buckley NM. Maturation of circulatory system in three mammalian models of human development. Comp Biochem Physiol A Comp Physiol. (1986) 83:1–7. 10.1016/0300-9629(86)90080-0 2868826

[41] Crissinger KD. Regulation of hemodynamics and oxygenation in developing intestine: insight into the pathogenesis of necrotizing enterocolitis. Acta Paediatr Suppl. (1994) 396:8–10. 10.1111/j.1651-2227.1994.tb13233.x 8086691

[42] Nankervis CA Reber KM Nowicki PT. Age-dependent changes in the postnatal intestinal microcirculation. Microcirculation. (2001) 8:377–87. 10.1111/j.1549-8719.2001.tb00185.x 11781811

[43] Boegehold MA. Endothelium-dependent control of vascular tone during early postnatal and juvenile growth. Microcirculation. (2010) 17:394–406. 10.1111/j.1549-8719.2010.00035.x 20618696 PMC3402360

[44] Chaaban H Stonestreet BS. Intestinal hemodynamics and oxygenation in the perinatal period. Semin Perinatol. (2012) 36:260–8. 10.1053/j.semperi.2012.04.006 22818546

[45] Crissinger KD Kvietys PR Granger DN. Developmental intestinal vascular responses to venous pressure elevation. Am J Physiol. (1988) 254:G658–63. 10.1152/ajpgi.1988.254.5.G658 3364567

[46] Nowicki PT Miller CE. Autoregulation in the developing postnatal intestinal circulation. Am J Physiol. (1988) 254(2 Pt 1):G189–93. 10.1152/ajpgi.1988.254.2.G189 3348374

[47] Nowicki PT Miller CE. Effect of O2 availability on intrinsic vascular response to venous pressure elevation in postnatal swine intestine. Am J Physiol. (1990) 258:G873–7. 10.1152/ajpgi.1990.258.6.G873 2360634

[48] Nowicki PT Miller CE Edwards RC. Effects of hypoxia and ischemia on autoregulation in postnatal intestine. Am J Physiol. (1991) 261(1 Pt 1):G152–7. 10.1152/ajpgi.1991.261.1.G152 1858883

[49] Nowicki PT Miller CE. Effect of increased tissue oxygen uptake on autoregulation in postnatal intestine. Am J Physiol. (1992) 263:G690–4. 10.1152/ajpgi.1992.263.5.G690 1443143

[50] Nowicki PT Miller CE. Regulation of capillary exchange capacity in postnatal swine intestine. Am J Physiol. (1993) 265:G1090–7. 10.1152/ajpgi.1993.265.6.G1090 8279560

[51] Nankervis CA Nowicki PT. Role of nitric oxide in regulation of vascular resistance in postnatal intestine. Am J Physiol. (1995) 268:G949–58. 10.1152/ajpgi.1995.268.6.G949 7611416

[52] Nakanishi T Gu H Abe K Momma K. Developmental changes in the contractile system of the mesenteric small artery of rabbit. Pediatr Res. (1997) 41:65–71. 10.1203/00006450-199701000-00010 8979291

[53] Nowicki PT. Effects of sustained flow reduction on postnatal intestinal circulation. Am J Physiol. (1998) 275:G758–68. 10.1152/ajpgi.1998.275.4.G758 9756507

[54] Reber KM Nowicki PT. Pressure and flow characteristics of terminal mesenteric arteries in postnatal intestine. Am J Physiol. (1998) 274:G290–8. 10.1152/ajpgi.1998.274.2.G290 9486182

[55] Nowicki PT. Effects of sustained low-flow perfusion on the response to vasoconstrictor agents in postnatal intestine. Am J Physiol. (1999) 276:G1408–16. 10.1152/ajpgi.1999.276.6.G1408 10362644

[56] Nankervis CA Nowicki PT. Role of endothelin-1 in regulation of the postnatal intestinal circulation. Am J Physiol Gastrointest Liver Physiol. (2000) 278:367–75. 10.1152/ajpgi.2000.278.3.G367 10712255

[57] Nankervis CA Schauer GM Miller CE. Endothelin-mediated vasoconstriction in postischemic newborn intestine. Am J Physiol Gastrointest Liver Physiol. (2000) 279:683–91. 10.1152/ajpgi.2000.279.4.G683 11005754

[58] Nankervis CA Dunaway DJ Nowicki PT. Determinants of terminal mesenteric artery resistance during the first postnatal month. Am J Physiol Gastrointest Liver Physiol. (2001) 280:G678–86. 10.1152/ajpgi.2001.280.4.G678 11254494

[59] Nankervis CA Dunaway DJ Miller CE. Endothelin ET(A) and ET(B) receptors in postnatal intestine. Am J Physiol Gastrointest Liver Physiol. (2001) 280:555–62. 10.1152/ajpgi.2001.280.4.G555 11254481

[60] Reber KM Mager GM Miller CE Nowicki PT. Relationship between flow rate and NO production in postnatal mesenteric arteries. Am J Physiol Gastrointest Liver Physiol. (2001) 280:G43–50. 10.1152/ajpgi.2001.280.1.G43 11123196

[61] Reber KM Su BY Reed Clark K Pohlman DL Miller CE Nowicki PT. Developmental expression of eNOS in postnatal swine mesenteric artery. Am J Physiol Gastrointest Liver Physiol. (2002) 283:G1328–35. 10.1152/ajpgi.00067.2002 12433665

[62] Su BY Reber KM Nankervis CA Nowicki PT. Development of the myogenic response in postnatal intestine: role of PKC. Am J Physiol Gastrointest Liver Physiol. (2003) 284:G445–52. 10.1152/ajpgi.00259.2002 12576303

[63] Su BY Reber KM Nankervis CA. Developmental expression of endothelin receptors in postnatal swine mesenteric artery. Pediatr Res. (2004) 56:359–65. 10.1203/01.PDR.0000134253.86014.B9 15240868

[64] Wendel M Kummer W Knels L Schmeck J Koch T. Muscular ETB receptors develop postnatally and are differentially distributed in specific segments of the rat vasculature. J Histochem Cytochem. (2005) 53:187–96. 10.1369/jhc.4A6474.2005 15684331

[65] Ayuso M Van Cruchten S Van Ginneken C Birthweight determines intestinal microvasculature development and alters endothelial nitric oxide synthase density in young piglets. Anat Histol Embryol. (2020) 00:1–8. 10.1111/ahe.12534 31995241

[66] Buckley NM Brazeau P Frasier ID Gootman PM. Circulatory effects of splanchnic nerve stimulation in developing swine. Am J Physiol. (1985) 248(1 Pt 2):H69–74. 10.1152/ajpheart.1985.248.1.H69 3970177

[67] Buckley NM Jarenwattananon M Gootman PM Frasier ID. Autoregulatory escape from vasoconstriction of intestinal circulation in developing swine. Am J Physiol. (1987) 252:H118–24. 10.1152/ajpheart.1987.252.1.H118 3812706

[68] Nowicki PT Miller CE Hayes JR. Effect of sustained mesenteric nerve stimulation on intestinal oxygenation in developing swine. Am J Physiol. (1991) 260(2 Pt 1):G333–9. 10.1152/ajpgi.1991.260.2.G333 1996651

[69] Hoang T V Choe EU Lippton HL Hyman AL Flint LM Ferrara JJ. Effect of maturation on alpha-adrenoceptor activity in newborn piglet mesentery. J Surg Res. (1996) 61:330–8. 10.1006/jsre.1996.0125 8656604

[70] Nowicki PT. Postnatal changes in gut hemodynamics: a possible role for substance P. Am J Physiol. (1998) 274:G1142–50. 10.1152/ajpgi.1998.274.6.G1142 9696715

[71] González-Luis G Fletcher AJW Moreno L Pérez-Vizcaíno F Blanco CE Villamor E Nitric oxide-mediated nonadrenergic noncholinergic relaxation of piglet pulmonary arteries decreases with postnatal age. J Physiol Pharmacol. (2007) 58:45–56. 17440225

[72] Coombs RC Morgan ME Durbin GM Booth IW McNeish AS. Abnormal gut blood flow velocities in neonates at risk of necrotising enterocolitis. J Pediatr Gastroenterol Nutr. (1992) 15:13–9. 10.1097/00005176-199207000-00003 1403445

[73] Martinussen M Brubakk AM Vik T Yao AC. Mesenteric blood flow velocity and its relation to transitional circulatory adaptation in appropriate for gestational age preterm infants. Pediatr Res. (1996) 39:275–80. 10.1203/00006450-199602000-00015 8825800

[74] Maruyama K Koizumi T Tomomasa T Morikawa A. Intestinal blood-flow veolicty in umcomplicated preterm infants during the early neonatal period. Pediatr Radiol. (1999) 29:472–7. 10.1007/s002470050621 10369910

[75] Yanowitz TD Yao AC Pettigrew KD Werner JC Stonestreet BS. Postnatal hemodynamic changes in very-low-birthweight infants. J Appl Physiol. (1999) 87:370–80. 10.1152/jappl.1999.87.1.370 10409597

[76] Maruyama K Koizumi T. Superior mesenteric artery blood flow velocity in small for gestational age infants of very low birth weight during the early neonatal period. J Perinat Med. (2001) 29:64–70. 10.1515/JPM.2001.009 11234619

[77] Havranek T Thompson Z Carver JD. Factors that influence mesenteric artery blood flow velocity in newborn preterm infants. J Perinatol. (2006) 26:493–7. 10.1038/sj.jp.7211551 16826195

[78] Papacci P Giannantonio C Cota F Latella C Semeraro CM Fioretti M. Neonatal colour Doppler ultrasound study: Normal values of abdominal blood flow velocities in the neonate during the first month of life. Pediatr Radiol. (2009) 39:328–35. 10.1007/s00247-008-1112-6 19189099

[79] Havranek T Miladinovic B Wadhawan R Carver JD. Factors that affect the postnatal increase in superior mesenteric artery blood flow velocity in very low birth weight preterm infants. J Perinat Med. (2012) 40:565–70. 10.1515/jpm-2011-0235 22945276

[80] Thompson A Silva CT Gork AS Wang D Ehrenkranz RA. Intestinal blood flow by doppler ultrasound: the impact of gestational age and time from first enteral feeding in preterm neonates. Am J Perinatol. (2014) 31:261–8. 10.1055/s-0033-1347365 23729284

[81] Gursoy T Imamoglu EY Ovali F Karatekin G. Effects of antenatal magnesium exposure on intestinal blood flow and outcome in preterm neonates. Am J Perinatol. (2015) 32:1064–9. 10.1055/s-0035-1548541 25825964

[82] Kocvarova L Mackovicova L Matasova K Zibolen M. The early postnatal blood flow characteristics in the superior mesenteric and coeliac arteries in late preterm neonates. J Matern Fetal Neonatal Med. (2018) 31:3027–32. 10.1080/14767058.2017.1362553 28760069

[83] Cortez J Gupta M Amaram A Pizzino J Sawhney M Sood BG. Noninvasive evaluation of splanchnic tissue oxygenation using near-infrared spectroscopy in preterm neonates. J Matern Fetal Neonatal Med. (2011) 24:574–82. 10.3109/14767058.2010.511335 20828232

[84] McNeill S Gatenby JC McElroy S Engelhardt B. Normal cerebral, renal and abdominal regional oxygen saturations using near-infrared spectroscopy in preterm infants. J Perinatol. (2011) 31:51–7. 10.1038/jp.2010.71 20539273 PMC3013378

[85] Patel AK Lazar DA Burrin DG O'Brian Smith E Magliaro TJ Stark AR. Abdominal near-infrared spectroscopy measurements are lower in preterm infants at risk for necrotizing enterocolitis. Pediatr Clin Care Med. (2014) 15:735–41. 10.1097/PCC.0000000000000211 25068253

[86] Bozzetti V Paterlini G Van Bel F Visser GHA Tosetti L Gazzolo D. Cerebral and somatic NIRS-determined oxygenation in IUGR preterm infants during transition. J Matern Fetal Neonatal Med. (2016) 29:443–6. 10.3109/14767058.2014.1003539 25604088

[87] Ledo A Aguar M Núñez-Ramiro A Saénz P Vento M. Abdominal near-infrared spectroscopy detects low mesenteric perfusion early in preterm infants with hemodynamic significant ductus arteriosus. Neonatology. (2017) 112:238–45. 10.1159/000475933 28704836

[88] Kuik SJ Van Zoonen AGJF Bos AF Van Braeckel KNJA Hulscher JBF Kooi EMW. The effect of enteral bolus feeding on regional intestinal oxygen saturation in preterm infants is age-dependent: a longitudinal observational study. BMC Pediatr. (2019) 19:404. 10.1186/s12887-019-1805-z 31684920 PMC6827212

[89] Nowicki PT Caniano DA Hammond S Giannone PJ Besner GE Reber KM. Endothelial nitric oxide synthase in human intestine resected for necrotizing enterocolitis. J Pediatr. (2007) 150:40–5. 10.1016/j.jpeds.2006.09.029 17188611

[90] Castillo RO Pittler A Costa F. Intestinal maturation in the rat: The role of enteral nutrients. J Parenter Enter Nutr. (1988) 12:490–5. 10.1177/0148607188012005490 3141647

[91] Hooper L V Wong MH Thelin A Hansson L Falk PG Gorden JI. Molecular analysis of commensal host-microbial relationships in the intestine. Science. (2001) 291:881–4. 10.1126/science.291.5505.881 11157169

[92] Chen YM Zhang JS Duan XL. Changes of microvascular architecture, ultrastructure and permeability of rat jejunal villi at different ages. World J Gastroenterol. (2003) 9:795–9. 10.3748/wjg.v9.i4.795 12679935 PMC4611452

